# Genomic insights into stroke recovery: cross-phenotype associations

**DOI:** 10.1093/braincomms/fcag322

**Published:** 2026-08-26

**Authors:** Chad Aldridge, Robynne Braun, Livia Parodi, Keith Lohse, Matthew A Edwardson, John W Cole, Arne G Lindgren, Fang-Chi Hsu, Keith Keene, Bradford Worrall, Jonathan Rosand, E Alison Holman, Steven C Cramer

**Affiliations:** Department of Neurology, University of Virginia, Charlottesville, VA 22908, USA; Department of Neurology, University of Maryland School of Medicine, Baltimore, MD 21201, USA; Department of Neurology, Center for Genomic Medicine, McCance Center for Brain Health, Massachusetts General Hospital, Boston, MA 02114, USA; Program in Physical Therapy, Department of Neurology, Washington University, St. Louis, MO 63108, USA; Department of Neurology and Rehabilitation Medicine, Georgetown University, Washington, DC 20007, USA; Department of Neurology, University of Maryland School of Medicine, Baltimore, MD 21201, USA; Department of Neurology, Baltimore Veterans Affairs Medical Center, Baltimore, MD 21201, USA; Department of Neurology, Skåne University Hospital, Lund 29189, Sweden; Department of Biostatistics and Data Science, Wake Forest University School of Medicine, Winston-Salem, NC 27101, USA; Department of Biology, East Carolina University, Greenville, NC 27858, USA; Department of Neurology, University of Virginia, Charlottesville, VA 22908, USA; Department of Public Health Sciences, University of Virginia, Charlottesville, VA 22908, USA; Department of Neurology, Center for Genomic Medicine, McCance Center for Brain Health, Massachusetts General Hospital, Boston, MA 02114, USA; Sue & Bill Gross School of Nursing and Department of Psychological Science, University of California, Irvine, Irvine, CA 92697, USA; Department of Neurology, University of California, Los Angeles, CA 90095-1769, USA; California Rehabilitation Institute, Los Angeles, CA 90067, USA

**Keywords:** GWAS, neuroplasticity, genomics, stroke, recovery

## Abstract

Stroke is a major cause of long-term disability with variable recovery. While clinical factors such as initial severity play a role, genetic factors are increasingly recognized as important contributors to stroke recovery. Genotype studies are generally focused on a single post-stroke behavioural domain, but some genes might relate to broad mechanisms of plasticity. This study therefore aimed to identify cross-phenotypic genetic variants associated across two or more stroke recovery domains. DNA from Stroke, Stress, Rehabilitation, and Genetics study participants was genotyped, resulting in 9 814 610 variants. In order to examine cross-phenotypic results, we first conducted genome-wide association studies on the six recovery domains: motor (grip force), cognition (Telephone Montreal Cognitive Assessment), depression (Patient Health Questionnaire-8), stress (Primary Care Post-Traumatic Stress Disorder Screen), functional status (Stroke Impact Scale-Activities of Daily Living), and disability (modified Rankin Scale 0–2 versus 3–6), some of which were tested longitudinally, yielding nine phenotypes. Models were adjusted for age, sex, initial severity (NIH Stroke Scale score), and ancestry. Cross-phenotype associations were identified by evaluating single nucleotide polymorphisms (SNPs) associated (*P* < 5e-5) with multiple phenotypes. To determine how these genetic variants may relate to biological mechanisms of recovery, we conducted gene enrichment analyses. Participants (*n* = 565, 59% male) had mild-moderate initial stroke severity (median acute NIH Stroke Scale score = 4). After accounting for the correlation structure among the nine phenotypes, we observed 319 cross-phenotypic SNPs, 3.45 times the expected number. Five of the cross-phenotypic SNPs were linked to genes relevant to neural development, function and plasticity, e.g. *ERICH1* (rs11778883-C), *FOX3* (rs55726768-G), *LIFR-AS1* (rs76401391-T), *RPS6KA2* (rs113518460-C) and *TUBGCP2* (rs147150392-C), as were enrichments in *RAB5–EEA1*, *CTNNA1–CTNNB1*, *CIN85–SH3GL2* and *ELMO1–DOCK2* complexes. Multiple gene enrichments were found, e.g. Stroke Impact Scale-Activities of Daily Living and Patient Health Questionnaire 8 at 3 months were enriched for *CREB* phosphorylation, which is important for long-term potentiation. We identified cross-phenotypic SNPs associated with multiple behavioural domains of stroke recovery. Some of these genes encode, or regulate, druggable proteins. These genetic factors are not well captured by clinical or neuroimaging assessments and so provide a unique window into stroke recovery. These findings, if validated, suggest that some genes may be broadly important to stroke recovery.

## Teaser

Aldridge *et al*. report that 319 genetic variants associated with multiple stroke recovery domains occur more than expected by chance. Genome-wide analyses across six recovery domains in 565 participants identify variants linked to neural plasticity genes, suggesting some genetic factors broadly influence stroke recovery and may encode druggable therapeutic targets.

## Graphical Abstract

**Graphical Abstract:**
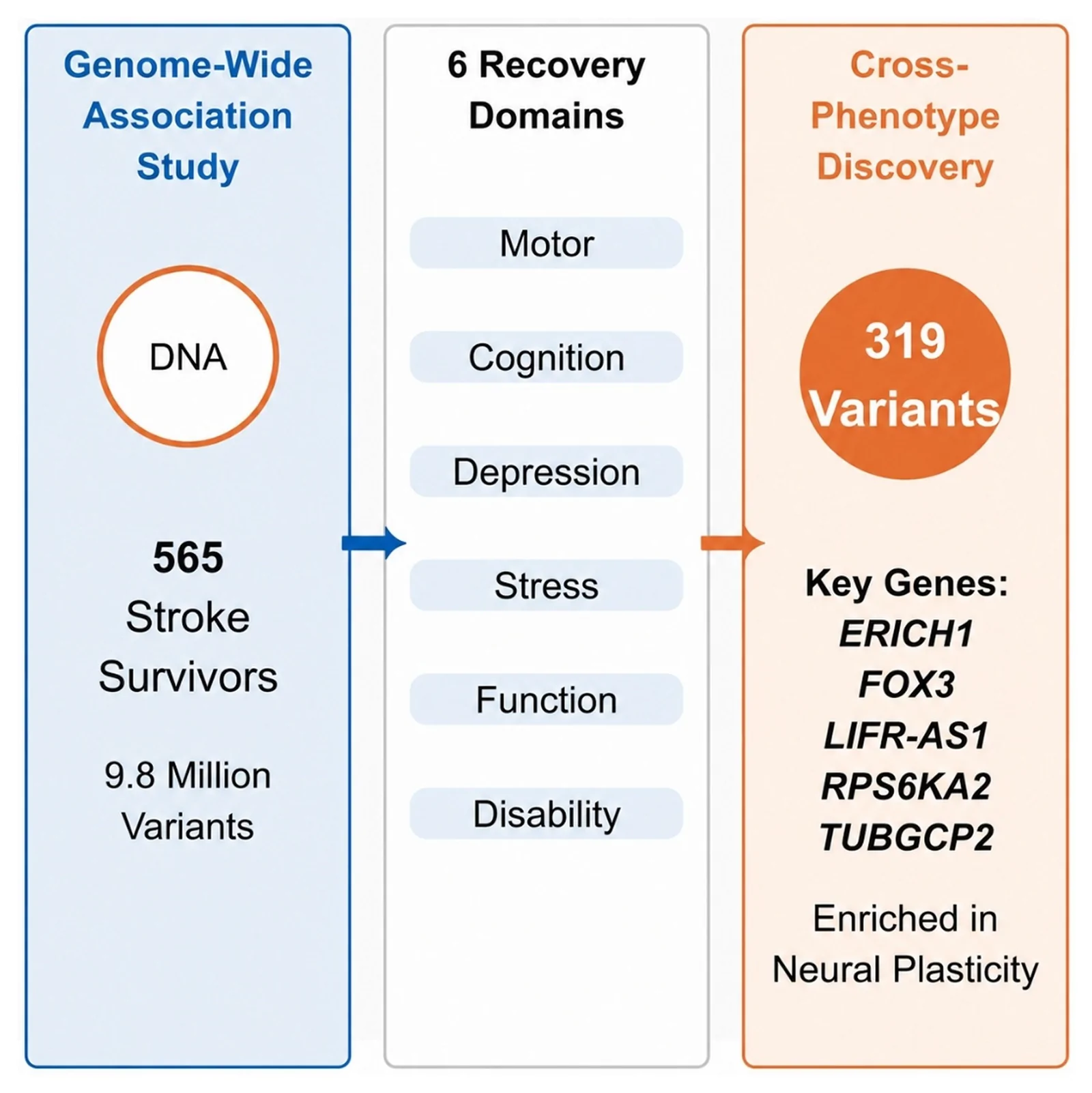

## Introduction

Stroke remains a significant contributor to long-term disability in the USA. In the USA, nearly 800 000 people suffer a stroke each year, with approximately half experiencing enduring disabilities that impede their daily activities.^1,2^ Globally, the impact is even greater, with 15 million people suffering strokes annually, leading to 5 million deaths and another 5 million facing permanent disabilities.^3,4^ The economic burden of stroke-related disabilities is profound, costing hundreds of billions of dollars annually in healthcare expenses, rehabilitation and lost productivity.^1,4^ This is expected to increase over time,^5^ underscoring the need for improved insights and treatments into stroke recovery.

Genomics offers a promising avenue to gain such insights into stroke recovery and its variation across patients, which are incompletely explained by clinical exam and neuroimaging studies.^6^ Through the identification of genetic predispositions and biomarkers, personalized medicine can be developed to tailor treatments and rehabilitation programmes to individual genetic profiles.

While the field of stroke risk genomics is relatively advanced, having identified over 30 genetic loci associated with stroke risk and developed predictive models such as genomic risk scores,^7,8^ stroke recovery genomics remains in its early stages. Stroke recovery genomics has begun to uncover the roles of candidate genetic variants, such as *BDNF* val^66^met, as they relate to stroke recovery.^6,9^ A small number of studies have made progress by using genome-wide association studies (GWAS) to study stroke recovery,^10-12^ but these suffer from the use of broad global outcome measures, which obscure recovery by combining multiple outcomes into a single value limiting granularity^13,14^

Despite these advancements, the field requires more extensive studies with larger and diverse cohorts to fully understand the genetic underpinnings of stroke recovery.^10,13-15^ Additionally, the traditional candidate gene studies of stroke recovery focus on specific genes based on preconceived hypotheses. As a result, these candidate gene hypotheses suffer from bias and high false positive rates, limiting reproducibility.^16^ Use of GWAS, on the other hand, provides an agnostic approach for evaluating the entire genome, allowing discovery of unexpected and novel genetic associations and biological mechanisms. In combination with bioinformatic tools and well-annotated and free-accessible databases, researchers can utilize GWAS to gain deeper biological insights via functional annotations, protein networks and pathway analyses.

To address these gaps in stroke recovery genomic discovery, we leveraged the STRONG (Stroke, Stress, Rehabilitation, Genetics) study^17^ to conduct multiple GWAS on stroke recovery for several specific behavioural domains,^18,19^ including motor, cognition and depression. The STRONG study is unique in the field of stroke recovery genomics as it observed participants from the acute phase in the hospital to 12 months post-stroke and collected a variety of detailed clinical measures at key recovery time points,^20^ with some collected repeatedly.

Despite the STRONG study’s strength for genetic discovery, it lacks the recommended sample size (*n* > 5000) for a GWAS of a single phenotype. To account for this, we applied a cross-phenotypic approach to find single nucleotide polymorphisms (SNPs) that are associated with multiple stroke recovery behavioural domains and timepoints. By incorporating several GWAS, each related to a separate behavioural phenotype, statistical power is increased by leveraging joint probabilities. This approach tests the hypothesis that some genes are related to broad mechanisms of plasticity and so correlate with stroke recovery across multiple behavioural domains.

## Materials and methods

### Stroke recovery cohort

A detailed description of the STRONG cohort can be found elsewhere.^17^ In summary, the STRONG study was an observational and prospective longitudinal cohort study that included participants 2–10 days after ischaemic stroke or intracerebral haemorrhage, aged 18 years,^3^ across 28 US acute care hospitals, between October 2016 and February 2020. Subjects were excluded if they had expected survival <1 year or moderate-severe pre-stroke disability (modified Rankin Scale (mRS) score >3).

Participants underwent four study visits at 2–10 days, 3 months, 6 months, and 12 months post-stroke, measuring multiple detailed stroke recovery phenotypes. These phenotypes correspond to important post-stroke recovery domains and biologically relevant time points (Table 1).^18,21^

**Table 1:** Behavioural domains studied

Domain	Measure	Phenotype	Visit 1 (*2 to 10 days*)	Visit 2 (*3 months*)	Visit 3 (*6 months*)	Visit 4 (*12 months*)
**Motor**	Grip strength	Delta grip (Visit 2–Visit 1)	x	x		
**Functional status**	SIS-ADL^22^	Total score		x		
**Disability**	mRS^23^	0–2 versus 3–6		x		x
**Depression**	PHQ8^24^	Total score		x	x	x
**Stress**	PTSD^25^	Total score			x	x
**Cognition**	tMoCA^26^	Total score				x

The tMoCA is the telephone version of the Montreal Cognitive Assessment; grip strength was measured by hand dynamometry; SIS-ADL is the score on the Stroke Impact Scale-Activities of Daily Living; mRS is the modified Rankin Scale score; PHQ8 is the 8-item Patient Health Questionnaire depression scale; and PTSD is the Primary Care PTSD Screen. Some of these six behavioural domains were studied longitudinally, yielding nine phenotypes.

### Informed consent

The study was approved by each participating site’s Internal Review Board, and each participant provided informed consent in line with the Declaration of Helsinki.

### Genotyping and quality control

DNA of 656 STRONG participants was extracted from saliva and genotyped using the Infinium Global Screening Array v1.0 (Illumina Inc., San Diego, CA). Genotypes were called with Illumina’s GenomeStudio v2.0.3 software to build 37. Markers were filtered based on GenomeStudio’s clustering algorithm’s metrics including a call rate greater than 97%. After the initial QC step, 594 samples passed. PLINK v1.9 (ref. ^24^) was used for additional QC. As a result, one sample was removed due to sex discrepancy and 26 due to having increased or decreased heterozygosity (≥3 SDs from the sample mean). Out of the 686 133 variants genotyped, 13 515 were removed due to greater than 3% missing across samples, 8383 variants due to deviation from Hardy–Weinberg equilibrium (*P* < 1e-6), and 167 605 variants due to a minor allele frequency (MAF) < 1%. After these QC steps, 565 samples and 496 630 variants remained. Principal component (PC) analysis was conducted on a subset of these variants not in linkage disequilibrium (--indep-pairwise 50 5 0.2) to account for population stratification. The top 10 PCs were calculated.

Genotype phasing and imputation were performed using single nucleotide variants without conflicting flipping (C/G or T/A) polymorphisms on autosomes. Imputation was performed on the Michigan Imputation Server^25^ using the Minimac3 algorithm and the Haplotype Reference Consortium^22^ (release 1.1) reference panel. Post-imputation data were filtered by imputation quality (*R*^2^ > 0.4) and MAF > 1%, yielding a total of 9 814 610 variants.

#### Stroke recovery phenotypes

Phenotypes were derived from six different stroke recovery domains (Table 1): (i) cognition, via the telephone-based Montreal Cognitive Assessment (tMoCA)^23,27^; (ii) motor, via isometric grip strength (ratio of affected/unaffected hand); (iii) depression, via the Patient Health Questionnaire Depression Scale-8-item (PHQ8)^28,29^; (iv) stress, via the Primary Care PTSD Screen (PTSD)^30^; (v) functional status, via the Stroke Impact Scale-Activity of Daily Living score (SIS-ADL)^31^; and (iv) disability, via the mRS.^18,32^

Some of these behavioural domains were measured longitudinally (Table 1), yielding nine phenotypes. For example, the grip strength ratio was measured 2–10 days post-stroke, then again at 3 months; the motor measure examined was the change from baseline to 3 months, i.e. delta grip. The PHQ8, and PTSD measures were also collected at least twice from 3 months to 12 months post-stroke. The repeated measurements allowed the investigation of time-dependent relationships between genetic variants and stroke recovery domains.

### Statistical analysis plan

#### Individual stroke recovery phenotype genome-wide association analysis

To examine cross-phenotypic associations, we first performed GWAS, using PLINK v2.0^10^ (--glm). Depending on the phenotype, linear or logistic regression models were fitted on imputed dosage data. Age at enrolment, first total NIHSS score on acute stroke admission, sex and the first 10 ancestry PCs were used as covariates in GWAS analyses, emulating a prior study.^11^ For individual genome-wide association analyses, we used traditional genome-wide association thresholds to consider a SNP significant with a *P-*value < 5e-8, and suggestive with a *P-*value < 5e-5.

#### Cross-phenotype genetic association analysis

**Figure 1:**
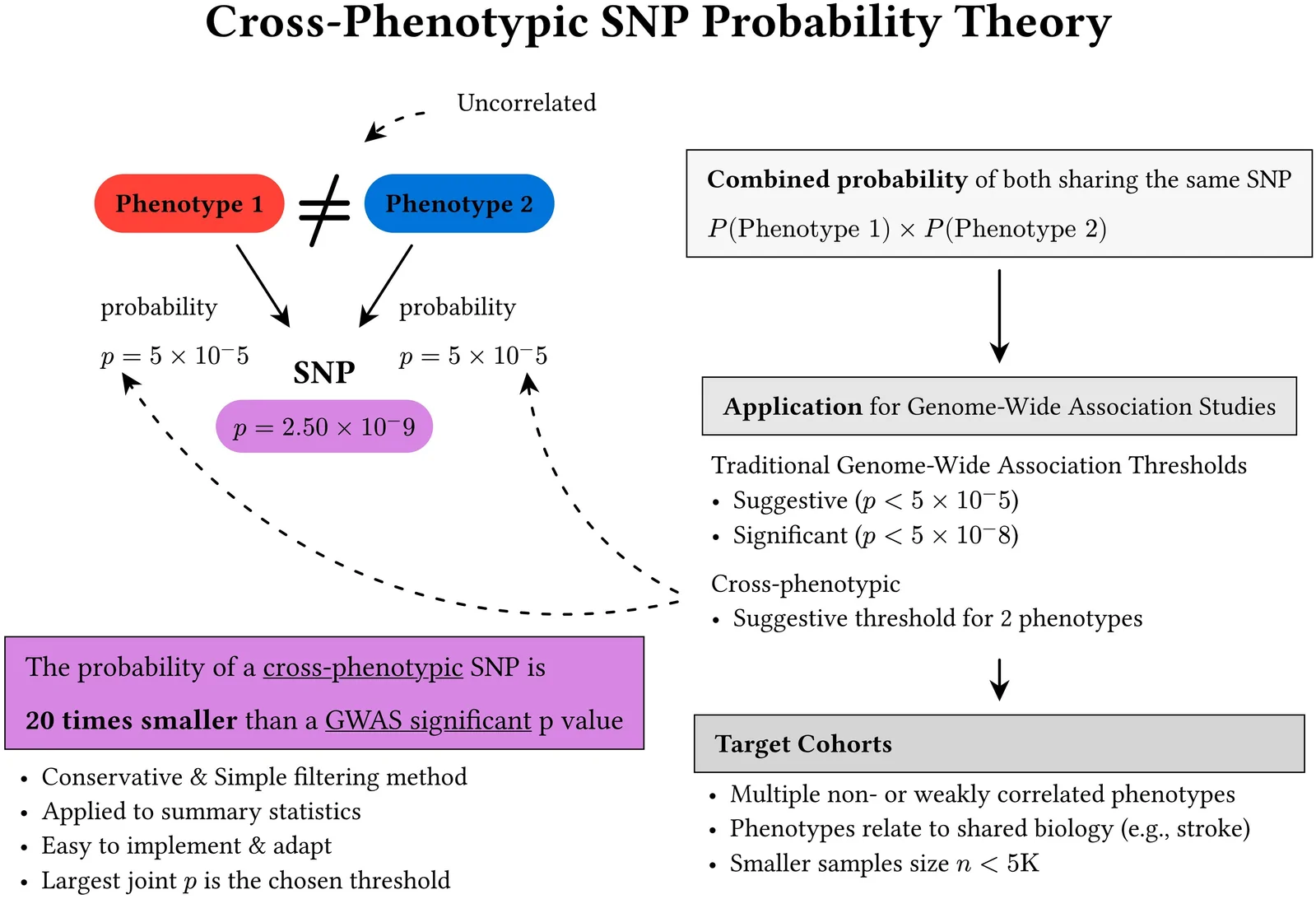
**Theoretical framework and cross-phenotyping technique to find genetic associations with multiple post-stroke deficit domains.** The probability of two events occurring is the product of their probabilities if these events are independent. In the Genome-Wide Association Study (GWAS) context, the probability that a single nucleotide polymorphism (SNP) is associated with two uncorrelated phenotypes (e.g. motor and cognitive impairment) is the product of the two *P-*values of the shared SNP. Choosing the traditional suggestive *P-*value threshold of 5e-5 for two or more phenotypes for a SNP to be considered cross-phenotypic equates to a probability of 2.50e-9. Note that this cross-phenotypic probability is 20 times smaller than the traditional GWAS significant threshold of 5e-8. If the phenotypes are perfectly correlated, then cross-phenotypic probability is the suggestive threshold, in this case being 5e-5. Therefore, even moderately correlated phenotypes will have a cross-phenotypic probability value between the chosen threshold and the product of the shared *P-*values, under the independence assumption.

**Figure 2:**
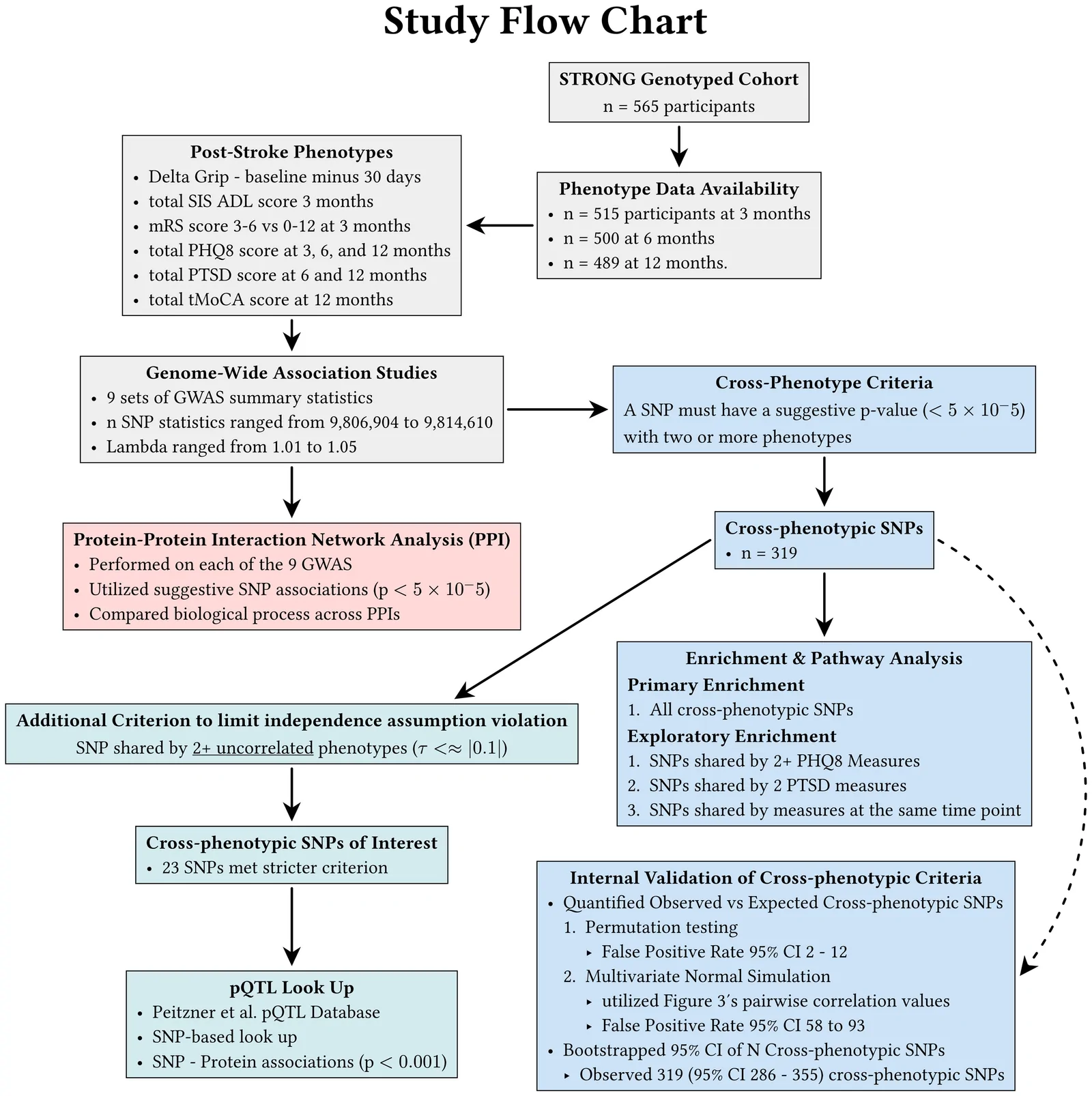
**Cross-phenotype stroke recovery GWAS schema.** The schema details the study design of utilizing the STRONG genotyped cohort and conducting genome-wide association studies (GWAS) on nine phenotypes (grey) using PLINK 2.0. The nine sets of GWAS summary statistics were used for constructing phenotype-specific protein–protein interaction (PPI) network analysis in red using the Search Tool for Retrieval of Interacting Genes/Proteins database (v.11.5, https://string-db.org/). We also applied our cross-phenotypic criterion to subset down to single nucleotide polymorphisms (SNP) that were suggestively associated with two or more phenotypes (blue). We conducted enrichment and pathway analyses with ‘gProfileR2’ R package by using these cross-phenotypic SNPs and conducted internal validation procedures (permutation testing and simulation) to determine if we observed more SNPs than expected due to change (blue). Lastly, we applied a stricter criterion which resulted in 23 cross-phenotypic SNPs of interest (green). To determine if these SNPs had a high likelihood of being cause, we did a protein Quantitative Trait Locus (pQTL) look-up with the Peitzner *et al*. pQTL database (green).

The aim of this study is to find cross-phenotypic SNPs. Identification of a cross-phenotypic SNP would support the hypothesis that some genes affect stroke recovery broadly, across several behavioural domains, and reflecting a shared biology influencing stroke recovery. We consider an SNP to be cross-phenotypic with stroke recovery if an SNP has a suggestive association (*P-*value < 5e-5) in two or more phenotypes. This equates to a joint probability of *P* < 2.5e-9, under the independence assumption (Fig. 1). This thresholding serves as a filtering step, reducing false positives and identifying SNPs for downstream analyses (Fig. 2). We chose the suggestive threshold due to the size of the genotyped STRONG cohort.

**Figure 3:**
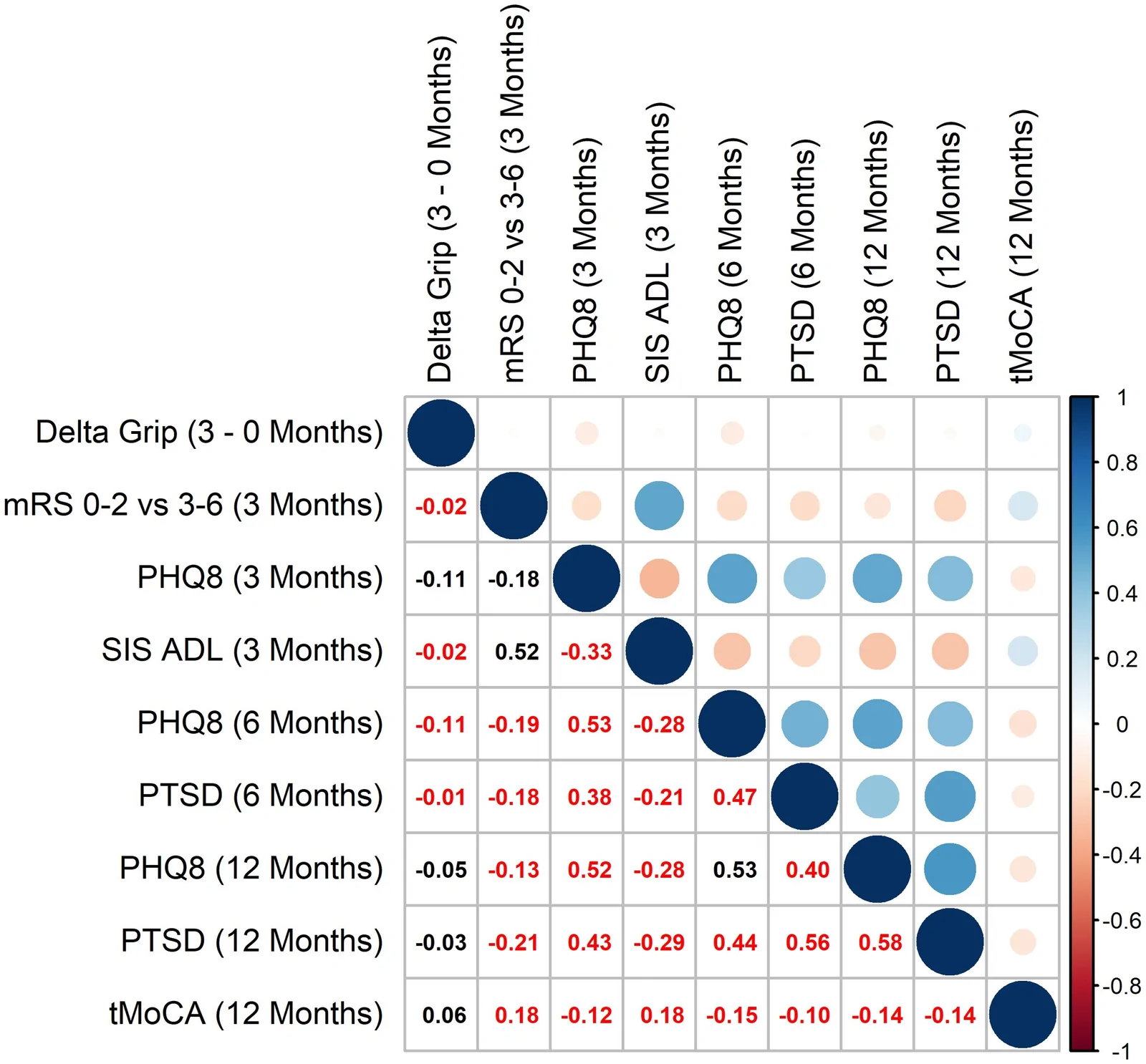
**Post-stroke clinical outcomes correlations.** Delta grip is the change in grip ratio (affected/unaffected hand) from baseline to 3 months; mRS, the modified Rankin Scale score; PHQ-8, the 8-item Patient Health Questionnaire Depression Scale; SIS-ADL, the score on the Stroke Impact Scale-Activities of Daily Living; PTSD, the Primary Care PTSD Screen; and tMoCA, the telephone version of the Montreal Cognitive Assessment. Numbers are tau correlation coefficients derived from Kendall’s method. They correspond to the legend scale a gradient from a negative correlation (red; −1), to no correlation (white; 0) to a positive correlation (blue; +1). Numbers in red are nominally significant with *P-*values < 0.05.

We compared the number of observed cross-phenotypic SNPs to the expected number, using two approaches. First, we conducted a permutation procedure in which the *P-*values were randomly shuffled across all sets. For each of 10 000 permutations, the number of *P-*values < 5e-5 was counted across the nine phenotype sets. This technique provides the expected number of cross-phenotypic SNPs due to the chance assuming all phenotypes are independent with all nine sets of GWAS summary *P-*values. Second, to account for the correlation structure among the nine stroke recovery phenotypes, we performed 10 000 simulations using a multivariate normal distribution and the correlation matrix from Fig. 3 to calculate the probability of a cross-phenotypic SNP under the null hypothesis. This technique generates simulated nine sets of 9 million *P-*values with the same pairwise correlation values observed in Fig. 3. We count the number of cross-phenotypic SNPs across the nine sets of 9 million simulated *P*-values, then repeat this process 10 000 times providing the median and 95% confidence interval of the count of cross-phenotypic SNPs due to chance. Thus, we have a reasonable estimate of the expected number of false positive cross-phenotypic SNPs based on our data.

To understand the potential causality of these cross-phenotypic SNPs, we conducted a protein quantitative trait loci (pQTL) look-up on cross-phenotypic SNPs to assess protein level associations. We utilized the publicly available pQTL database created by Peitzner *et al*.^33^ We considered SNP*–*protein associations to be of interest at *P* < 0.001.

##### Gene-based analysis of cross-phenotypic SNPs

Next, we conducted gene enrichment analyses among all cross-phenotypic SNPs using the ‘gProfileR2’ R package.^34^ This software utilizes multiple biological databases (e.g. WikiPathways, Gene Ontology, Kyoto Encyclopedia of Genes and Genomes) to find terms significantly enriched. Each term receives a hypergeometric *P-*value, on which we performed a false-discovery rate correction (FDR < 0.05).

We conducted exploratory gene enrichments among pairwise cross-phenotypic combinations (e.g. tMoCA and PTSD at 12 months post-stroke) as well as for SNPs repeatedly suggestive (*P* < 5e-5) within the same phenotype (e.g. PHQ8) across time (e.g. 3-, 6-, and 12 months). This allows the investigation of which enrichment terms are driven by specific follow-up time points and within repeated measures (e.g. PHQ8 at 3, 6, and 12 months).

##### Protein–protein interaction network of individual phenotypes

Next, we took the suggestively associated SNPs (*P* < 5e-5) for each phenotype and annotated them with their known genes. These gene lists were utilized to create protein–protein interaction (PPI) networks and to perform gene ontology (GO) analyses. This approach provided two advantages: (i) the reduction of the false positive likelihood rate and (ii) the mapping genetic associations to biological mechanisms.

To evaluate the biological relevance of genetic variants associated with recovery phenotypes in GWAS, we constructed a PPI network using the Search Tool for Retrieval of Interacting Genes/Proteins database (v.11.5, https://string-db.org/).^35^ The resulting list of unique gene IDs for single nucleotides (SNPs) within coding regions of the genome was used to retrieve a corresponding set of proteins, represented as nodes in the PPI network. For edge construction between the nodes, we specified an interaction score of 0.4 (moderate confidence) for all available evidence channels. The resulting PPI networks were visualized using the Cytoscape^36^ R package.

To further examine how identified genetic variants and their corresponding proteins relate to the biology of recovery, we then conducted GO enrichment analysis on the derived PPI networks, focused on cellular localization and biological processes terms (v. 2022-06-01).^13^ All PPI and GO term enrichment analyses were conducted in R using Bioconductor (R v4.3.0, BiocManager v1.30.21). Statistical significance for GO term enrichment was computed using a hypergeometric test and corrected for multiple testing using the Benjamini and Hochberg method.^37^

## Results

There were 565 participants with genotype data that passed the quality controls steps. Outcome data were available in 515 participants at 3 months, 500 at 6 months, and 489 at 12 months. The primary loss-to-follow-up reasons were mortality and inability to travel back to the enrolment site at 3 months. Stroke severity (first total NIHSS score on acute stroke admission), age, and gender did not differ between those that were observed versus lost-to-follow-up at 3 and 6 months; the same was true at 12 months, except those that were lost-to-follow-up had higher baseline NIHSS total scores [5 (2–10) versus 4 (2–8), *P* = 0.006]. Table 2 contains participant characteristics.

**Table 2:** Cohort demographics and phenotype measures of central tendency

	Genotyped				
Characteristic	No *N* = 198	Yes *N* = 565	Overall *N* = 763	*P*-value^a^	*q*-value^b^
**Age**	65 (55, 76)	64 (53, 73)	64 (54, 74)	0.2	0.5
**Gender**				0.023	0.2
Male	104 (53%)	344 (61%)	448 (59%)		
Female	93 (47%)	221 (39%)	314 (41%)		
Other	1 (0.5%)	0 (0%)	1 (0.1%)		
**Race**				<0.001	0.01
European	120 (61%)	409 (73%)	529 (69%)		
African	30 (15%)	97 (17%)	127 (17%)		
Asian	28 (14%)	13 (2.3%)	41 (5.4%)		
Mixed ancestry	9 (4.5%)	28 (5.0%)	37 (4.9%)		
Other	6 (3.0%)	10 (1.8%)	16 (2.1%)		
Indegenous	3 (1.5%)	5 (0.9%)	8 (1.0%)		
Pacific Islander	2 (1.0%)	2 (0.4%)	4 (0.5%)		
*N*-Missing	0	1	1		
**Ethnicity**				0.7	0.8
Non-Hispanic	169 (85%)	487 (86%)	656 (86%)		
Hispanic	26 (13%)	73 (13%)	99 (13%)		
Unknown	3 (1.5%)	5 (0.9%)	8 (1.0%)		
**BMI**	29 (25, 33)	29 (25, 34)	29 (25, 34)	0.3	0.5
*N*-Missing	2	7	9		
**Infarct side**				0.4	0.6
Right	109 (58%)	270 (53%)	379 (54%)		
Left	69 (37%)	219 (43%)	288 (41%)		
Bilateral	9 (4.8%)	23 (4.5%)	32 (4.6%)		
*N*-Missing	11	53	64		
**Hypercholesterolaemia**	93 (47%)	282 (50%)	375 (49%)	0.5	0.7
*N*-Missing	0	1	1		
**Diabetes**	51 (26%)	178 (32%)	229 (30%)	0.13	0.4
**A-Fib**	40 (20%)	83 (15%)	123 (16%)	0.07	0.4
**Prior stroke**	39 (20%)	106 (19%)	145 (19%)	0.8	0.8
*N*-Missing	0	2	2		
**Baseline NIHSS total score**	5.0 (2.0, 10.0)	4.0 (2.0, 8.0)	4.0 (2.0, 9.0)	0.3	0.5
*N*-Missing	7	26	33		
**Delta grip V1–V2**	0.08 (−0.05, 0.23)	0.09 (−0.04, 0.27)	0.09 (−0.04, 0.26)	0.8	0.8
*N*-Missing	97	229	326		
**mRS 3–6 versus 0–1 V2**	81 (65%)	288 (69%)	369 (68%)	0.4	0.6
*N*-Missing	73	148	221		
**Total SIS-ADL score V2**	35 (25, 39)	35 (26, 39)	35 (26, 39)	0.5	0.7
*N*-Missing	83	169	252		
**PHQ8 score V2**	5.0 (2.0, 10.0)	5.0 (2.0, 9.0)	5.0 (2.0, 9.0)	0.4	0.6
*N*-Missing	81	171	252		
**PHQ8 score V3**	5.0 (2.0, 10.0)	4.0 (1.0, 10.0)	4.0 (1.0, 10.0)	0.088	0.4
*N*-Missing	87	182	269		
**PHQ8 score V4**	5.0 (1.0, 10.0)	4.0 (1.0, 9.0)	4.0 (1.0, 9.0)	0.5	0.7
*N*-Missing	92	186	278		
**PTSD score V3**	3.0 (1.0, 6.0)	2.0 (0.0, 6.0)	2.0 (0.0, 6.0)	0.1	0.4
*N*-Missing	90	190	280		
**PTSD score 4V**	3.0 (0.0, 6.0)	2.0 (0.0, 6.0)	2.0 (0.0, 6.0)	0.12	0.4
*N*-Missing	91	184	275		
**tMoCA score V4**	18.0 (14.0, 19.0)	18.0 (15.0, 20.0)	18.0 (15.0, 20.0)	0.8	0.8
*N*-Missing	93	186	279		

Median (Q1, Q3); *n* (%).

^a^Wilcoxon rank sum test; Fisher’s exact test for count data with simulated *P*-value (based on 2000 replicates); Pearson’s chi-squared test.

^b^Benjamini and Hochberg correction for multiple testing.

V1: Baseline 5 to 10 days post-stroke. V2: 3 months post-stroke. V3: 6 months post-stroke. V4: 12 months post-stroke. Delta grip is the change in the grip strength ratio. SIS-ADL is the Stroke Impact Scale ADL subsection. mRS is the modified Rank Scale score. PHQ8 is the Patient Health Questionnaire-8 measure of depression severity. PTSD is the PTSD checklist for Primary Care, a 4-item measure of anxiety.

Some of the stroke recovery phenotypes were correlated; see Fig. 3. Delta grip (0–3 months) and tMoCA score at 12 months were uncorrelated with all other measures. In contrast, the SIS-ADL, mRS, PHQ8, and PTSD scores were moderately correlated (Kendall’s |tau| = 0.4–0.58) from the same visit time point. We observed less autocorrelation than expected for the PHQ8 (tau = 0.53 between 3 and 6 months; tau = 0.53 between 6 and 12 months) and PTSD (tau = 0.56 between 6 and 12 months) measures.

### Genome-wide association studies across phenotypes

We performed genome-wide association analysis on nine phenotypes, which span multiple stroke recovery domains (Table 3). The number of SNP associations tested ranged from 9 806 904 to 9 814 610. The genomic inflation lambda value ranged from 1.00 to 1.05. Even though delta grip had an acceptable lambda value, it displayed notable inflation in the number of genome-wide suggestive (*P* < 5e-5) and significant (*P* < 5e-8) SNPs and by visual inspection of its QQ-plot (Supplementary File 1). Further investigation revealed that this inflation was driven by SNPs with a MAF <5%. Supplementary File 1 contains the Manhattan and QQ plots for each phenotype. Each set of summary statistics is publicly available at the Cerebrovascular Disease Knowledge Portal. Specifically, PTSD at 6 and 12 months and the tMoCA had acceptable lambda values £1.05 and genome-wide significant SNP associations totalling 6, 32, and 5, respectively; for the other five GWAS, lambda values were acceptable but no genome-wide significant SNP associations were identified. For PTSD, none of the genome-wide significant SNPs (*n* = 32) could be replicated at 6 and 12 months. Some of these SNPs are found within the *DLGAP1* (post-synaptic scaffold of neuronal cells), *PET112* aka *GATB* (mitochondrial function), *PDGRFL* (tumour suppression) and *SUSD3* (oestrogen-dependent cell proliferation) genes, which may have roles in stroke recovery. Others are located within 50kbp of *AC09196.1* (lincRNA), *CTB-27N1.1* (lincRNA) and the pseudogenes: *HNRNPABP1*, *RP11-29BP.2*, *RP11-656G20.1* and *RP11-378A12.1*. None of the five significant SNPs associated with the tMoCA at 12 months were located near a gene.

**Table 3:** GWAS meta-stats for the nine phenotypes

Phenotype	*N* Observations	Genomic inflation (Lambda)	*N* SNPs analysed	*N* Suggestive SNPs	*N* Significant SNPs
**Delta grip ratio from baseline to 3 months**	335	1.02	9 814 610	6516	147
**mRS 0–2 versus 3–6 at 3 months**	416	1.01	9 813 940	99	0
**PHQ8 at 3 months**	393	1.01	9 814 610	745	0
**SIS-ADL at 3 months**	395	1.02	9 814 610	834	0
**PHQ8 at 6 months**	382	1.00	9 814 610	503	0
**PTSD at 6 months**	374	1.05	9 814 610	839	6
**PHQ8 at 12 months**	378	1.02	9 814 610	629	0
**PTSD at 12 months**	380	1.03	9 814 610	1691	32
**tMoCA at 12 months**	378	1.03	9 814 610	1508	5

Genotyping was called to GRChr37. Delta grip ratio refers to the ratio of grip strength (kg) in the (affected/unaffected) hand; mRS, the modified Rankin Scale score; SIS-ADL, score on the Stroke Impact Scale-Activity of Daily Living scale; tMoCA, the telephone version of the Montreal Cognitive Assessment. Suggestive and significant SNPs are defined as having a *P-*value less than 5e-5 and 5e-8, respectively. The mRS 0–2 versus 3–6 phenotype-derived *P-*values from a *F*-statistics derived from logistic regression models, while all other phenotypes derived *P-*values from *t*-statistics from linear regression models.

### Cross-phenotypic results

#### Observed versus expected number of cross-phenotypic SNPs

**Figure 4:**
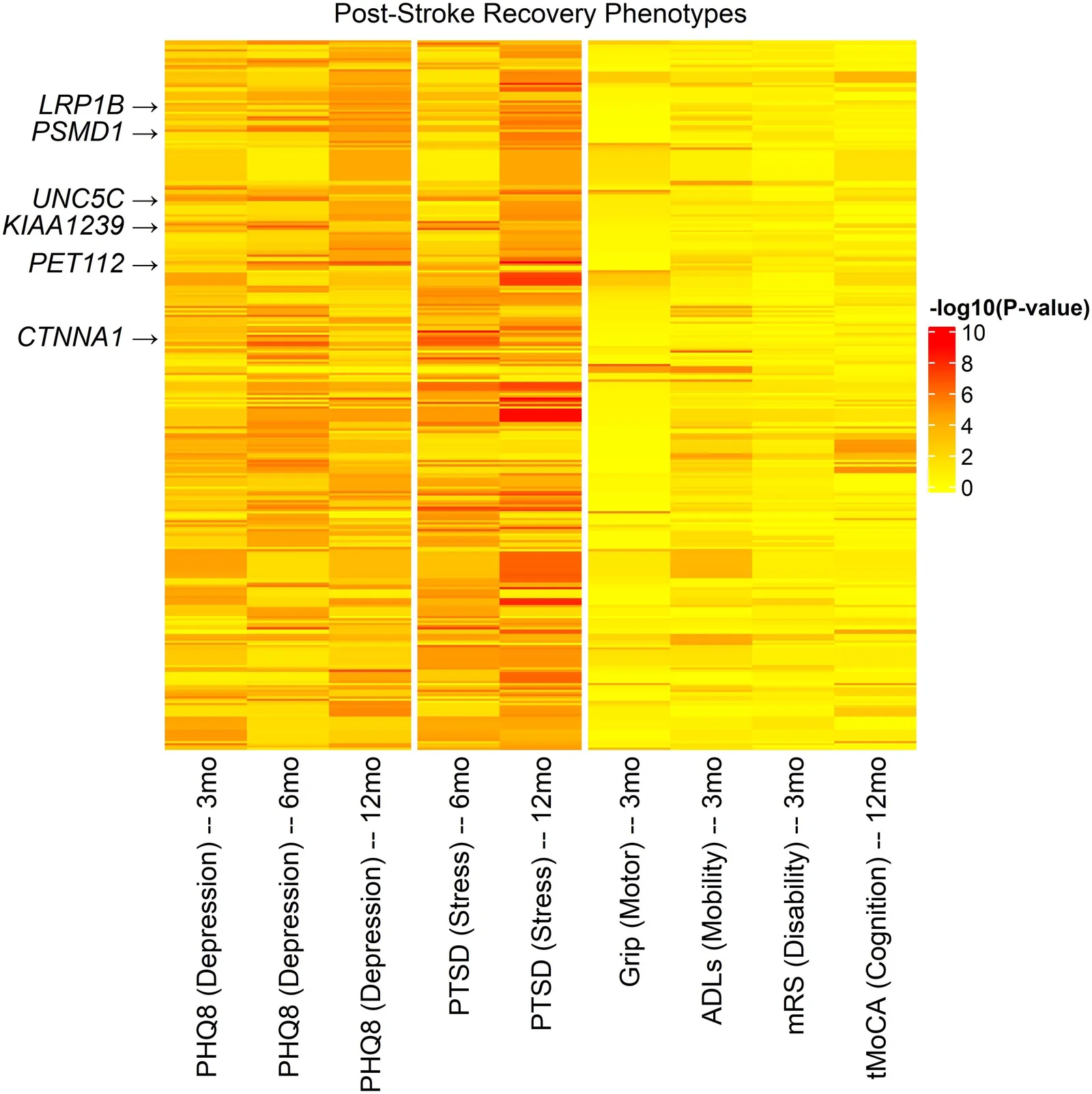
**.Phenotype-SNP association patterns for SNP suggestive of two or more phenotypes.** This is a heatmap showing horizontal line segments representing the −log10 of the *P*-value of each cross-phenotypic single nucleotide polymorphism (rows) from the nine phenotypes (columns). The heatmap legend shows the genome-wide association study (GWAS) −log10 of the *P*-value via a colour gradient from yellow to orange to red. The darker the red the colour, the more significant the SNP-phenotype association. A −log10 of the *P*-value of 4.3 is equivalent to a genome-wide suggestive association (*P* < 5e-5); a value of 7.3, to a genome-wide significant association (*P* < 5e-8). The *P-*values were derived from the linear (*t*) / logistic (*F*) statistics calculated from the phenotype-specific GWAS regression models. Each column corresponds to a post-stroke recovery measure. From left to right, PHQ8 is the Patient Health Questionnaire depression scale measure of depression. The Primary Care PTSD Screen (PTSD) is a measure of stress. Grip refers to the change in isometric grip strength from 3 months to baseline. ADLs refer to the total score of the Stroke Impact Scale-Activities of Daily Living (SIS-ADL) sub-items, a measure of functional status. mRS, a measure of disability, is the modified Rankin Scale score dichotomized as 0–2 (good) versus 3–6 (poor) outcome. tMoCA is the telephone version of the Montreal Cognitive Assessment. The far-left annotation lists genes that contain a SNP suggestively associated with three or more phenotypes (e.g. CTNNA1).

We observed 319 (2.5%) cross-phenotypic SNP shared by two or more stroke recovery phenotypes (Supplementary Table 1). Figure 4 displays a heatmap of the −log10 (*P*-values) derived from the nine GWAS for each cross-phenotypic SNP. The mood-related domains (stress and depression) are much more genetically similar than cognition, disability, functional status and motor domains. The genes listed on the far left of the heatmap contain SNPs that are suggestive in three or more phenotypes. For example, the *CTNNA1* gene contains rs114875370, which is suggestive for PHQ8 at 3 and 6 months, and for PTSD at 6 and 12 months; *CTNNA1* is expressed in axons and is important for cell interactions.

To understand how the cross-phenotypic approach impacts each set of GWAS summary statistics, we calculated the pairwise proportions that a single SNP is suggestively associated (*P* < 5e-5) across stroke recovery phenotypes and time. Several pairwise combinations failed to observe at least one cross-phenotypic SNP, suggesting a proportion of statistical noise was removed. For example, the delta grip GWAS exhibited right-tailed inflation of *P-*values, resulting in 147 significant and 6516 suggestive SNPs (Supplementary File 1). Our cross-phenotypic definition reduced the number of SNPs down to 10 among the pairwise combinations of delta grip with SIS-ADL (*n* = 4) at 3 months, PTSD (*n* = 1) at 6 months, and PHQ8 (*n* = 1), PTSD (*n* = 4) and tMoCA (*n* = 1) at 12 months post-stroke.

To determine how much the observed cross-phenotypic SNP probabilities differ from the theoretical frame in Fig. 1, we compared the number of cross-phenotypic SNPs (*n* = 319) to the expected number derived from two different approaches. First, we conducted a permutation procedure in which the *P-*values were randomly shuffled across all sets. For each permutation, the number of *P-*values less than 5e-5 was counted across the nine sets. The procedure performed 10 000 permutations resulting in an average (95% CI) count of 6.5 (2–12) cross-phenotypic SNPs. In other words, based on observed *P-*values of the nine GWAS sets, we expect between 2 and 12 cross-phenotypic SNPs due to random chance alone. This suggests that this study’s 319 cross-phenotypic SNPs are significantly enriched in number.

The second approach aimed to account for the correlation structure among the nine stroke recovery phenotypes. We performed 10 000 simulations using a multivariate normal distribution and the correlation matrix in Fig. 3 to calculate the probability of a cross-phenotypic SNP under the null hypothesis. We observed a two-tailed mean (95% CI) probability of 7.6e-6 (5.9e-6–9.4e-6) equating to a 95% CI range of 58 to 93 cross-phenotypic SNPs due to chance. In other words, we expected an upper value of 93 false positive cross-phenotypic SNPs. We observed 3.45 (319/93) times more cross-phenotypic SNPs compared to the upper bound of the expected number of false positive cross-phenotypic SNPs. Additionally, we bootstrapped 10 000 times the 9 814 610 SNPs to count the number of cross-phenotypic SNPs, which resulted in a 95% CI from 286 to 355. Because the bootstrapped 95% CI does not overlap either the permutation or simulation 95% CI, we observed significantly more cross-phenotypic SNPs than the number of expected false positive ones.

#### Cross-phenotypic SNPs of interest

**Figure 5:**
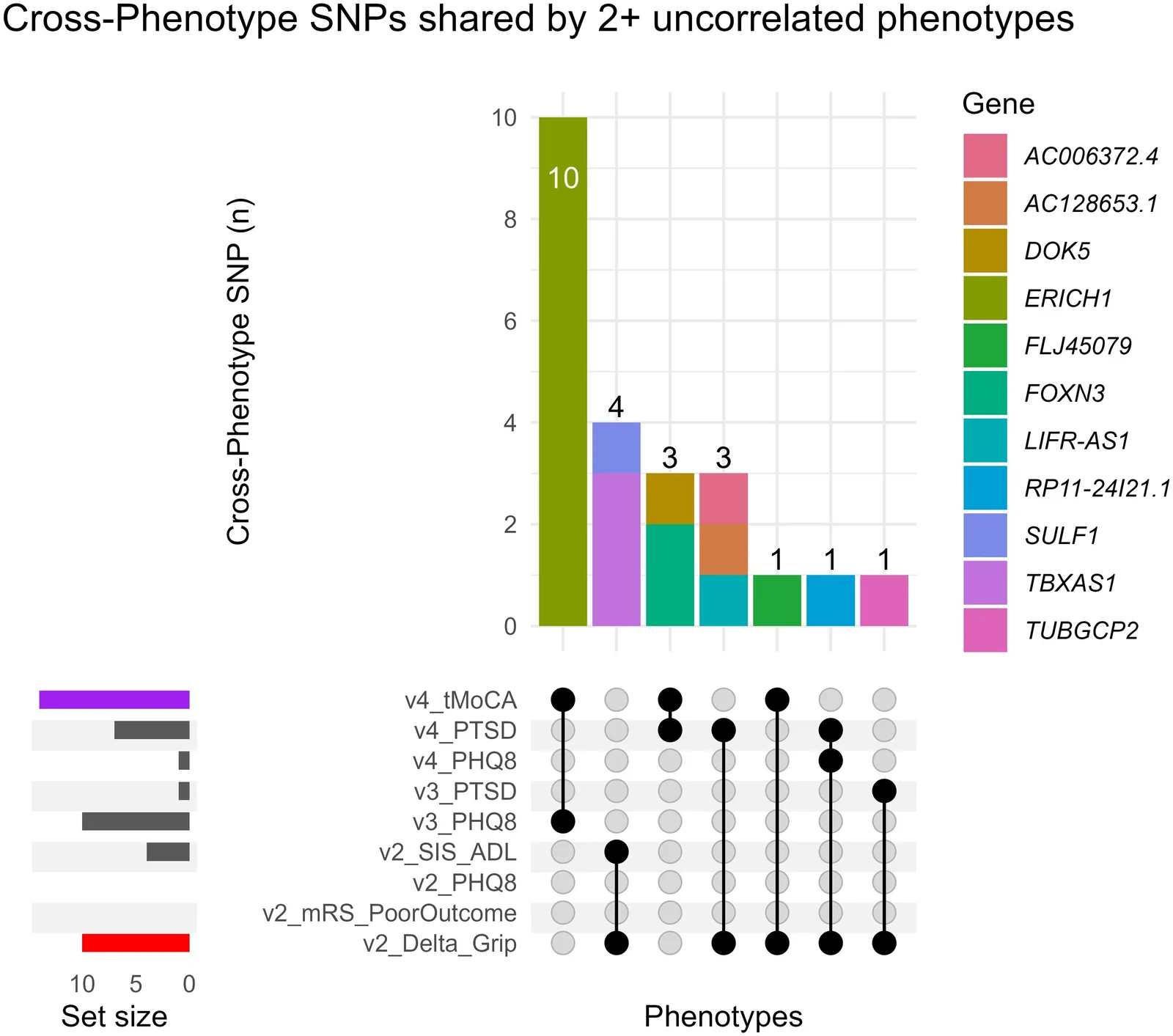
**Shared genetic variants linking motor recovery, mood, and cognition after stroke.** This is an UpSet plot showing the counts of SNPs in the top histogram for each combination of phenotypes that share at least one SNP. The top histogram shows the count of SNPs in each column with colours corresponding to their annotated gene symbols. For example, ERICH1, a protein-coding gene, contains 10 cross-phenotypic SNPs shared between the v4_tMoCA and v3_PHQ8 phenotypes. The histogram on the bottom left shows the count of cross-phenotypic SNPs related to each phenotype (e.g. v2_Delta_Grip has 10 out of the 23 cross-phenotypic SNPs). The red and purple bars are highlighting the v2_Delta_grip and v4_tMoCA phenotypes, which represent the spectrum of stroke domains from motor to cognition. The black dots and segments of the bottom matrix plot show each unique combination of phenotypes that share a SNP, which corresponds to the top histogram’s bins (e.g. the second matrix column from the right shows three black dots representing one SNP shared by v2_Delta_Grip, v4_PHQ8, and v4_PTSD phenotypes found in the RP11-24I21.1 pseudo-gene). The v[2,3,4] prefix refers to the visit timepoint with 2, 3 and 4 equating to 3-, 6- and 12-month post-stroke. PHQ8 is the Patient Health Questionnaire depression scale measure of depression. The Primary Care PTSD Screen (PTSD) is a measure of stress. Delta_Grip refers to the change in grip strength ratio (affected/unaffected) from baseline to 3 months. ADLs refer to the total score of the Stroke Impact Scale-Activities of Daily Living (SIS-ADL) sub-items, a measure of functional status. mRS, a measure of disability, is the modified Rankin Scale score dichotomized as 0–2 (good) versus 3–6 (poor) outcome. tMoCA is the telephone version of the Montreal Cognitive Assessment.

Of the 319 cross-phenotypic SNPs, 23 SNPs were suggestively associated (*P* < 5e-5) with two or more uncorrelated phenotypes (Supplementary Table 2; Fig. 5). Additionally, we cross-referenced each one with the pQTL database created by Peitzner *et al.*^33^ to determine if there is a cross-phenotypic SNP–protein expression association (Supplementary Table 3). Of the 23 cross-phenotypic SNPs, we highlight nine examples below.

The SNP rs138829971-C was cross-phenotypic with delta grip at 3 months and PHQ8 and PTSD at 12 months, thus overlapping motor, depression and stress domains (Fig. 5). It is located near a long intergenic non-protein-coding RNA (lincRNA), *RP11-24I1.1*. Delta grip had another cross-phenotypic SNP (rs76401391-T) with PTSD at 12 months located within the *LIFR-AS1* gene, antisense to the *LIFR* gene. PHQ8 at 3 months shared rs113518460-C (located within *RPS6KA2*) with SIS-ADL score at the same post-stroke time point (Supplementary Table 2). We observed 10 shared SNPs between PHQ8 score at 6 months and tMoCA scores at 12 months, spanning the cognition and depression domains (Fig. 5). All 10 are located within the protein-coding Glutamate Rich 1 (*ERICH1*) gene. Several had pQTL associations. For example, the rs11778883-C variant was associated with (Supplementary Table 3) phosphoglycerate kinase 2 (*PGK2*; *P* = 3e-4), cerebral dopamine neurotrophic factor (*CDNF*; *P* = 8e-4), neuronal growth regulator 1 (*NEGR1*; *P* = 8e-4) and polyglutamine-binding protein (*PQBP1*; *P* = 9e-4) levels.

Between tMoCA and delta grip phenotypes, we observed one shared SNP, rs149456987-T, which is a cis-variant of the *LOC105371908* gene. It is a long non-coding RNA. In relation to PTSD at 6 months, there is a shared SNP with delta grip at 3 months, rs147150392-C. This SNP is within the *TUBGCP2* gene and has the same direction effect between PTSD scores and delta grip indicating more severe symptoms and a worse grip ratio for each allele (Supplementary Table 2).

The PTSD and tMoCA scores at 12 months share rs55726768-G. This SNP is located within the *FOXN3* gene (Supplementary Table 2). Unlike the other previously mentioned cross-phenotype associated genes, *FOXN3* acts as a transcriptional repressor and is likely involved in stopping cell cycles that damage DNA.^38^ It also has a role in glucose utilization.^39,40^

While the PHQ8 and PTSD measures at 12 months post-stroke have a correlation equalling 0.58, we noted that one of the cross-phenotypic SNPs (rs142356140-T) located within the *RAB5C* gene had a highly significant pQTL association with cytosolic 5′-nucleotidase III-like protein (*P* = 1.17-e17) also known as *NT5C3L*. The other cross-phenotypic SNPs had pQTL associations with interleukin-7 receptor subunit alpha (IL-7 Ra), neuregulin-1 sensory and motor neuron-derived factor isoform (*SMDF*), signal transducer and activator of transcription 3 (STAT3), thrombopoeitin receptor, and cyclin-dependent kinase 2-interaction protein (*CINP*); see Supplementary Table 2.

### Enrichment analyses

To determine if there is a consistent theme of biological mechanisms or processes, we examined gene enrichment analyses on the cross-phenotype SNPs and each PPI network.

#### Cross-phenotypic gene enrichment results

Among the 319 cross-phenotypic SNPs, we observed functional gene enrichment across the comprehensive resource of mammalian protein complexes (CORUM), GO, and Reactome (REAC) terms (Supplementary Table 4). The top five significant CORUM enrichments after FDR correction were *RAB5–EEA1* complex, cell–cell junction complex (*ARHGAP10–CTNNA1*), *CTNNA1–CTNNB1* complex, *CIN85–SH3GL2* complex and the *ELMO1–DOCK2* complex. The top five biological process GO term enrichments were system development, cell differentiation, cellular developmental process, multicellular organismal process and developmental process. For cellular component GO terms, cell junction organization, cell junction, synapse, cell projection and plasma membrane region were the top five enriched terms. There were two REAC enrichments, including *CREB1* phosphorylation through NMDA receptor-mediated activation of RAS signalling and signalling by neurotrophin tyrosine kinase receptors (NTRKs).

For cross-phenotypic SNPs aligned at the same post-stroke time point, we found gene enrichments between the SIS-ADL (functional status) and PHQ8 (depression) measures at 3 months post-stroke, between PTSD (stress) and PHQ8 at 6- and 12-months post-stroke, and between PTSD and tMoCA at 12 months post-stroke (Supplementary Table 5). Mostly, notable between SIS-ADL and PHQ8 phenotypes (Fig. 4; *r* = −0.33), we observed enrichment of the KEGG pathway ‘insulin resistance’ (KEGG:04931) and the REAC ‘CREB phosphorylation’ and ‘RSK activation’ terms. For the tMoCA and PTSD at 12 months post-stroke, there was an enrichment of the brain-derived neurotrophic factor (*BDNF*) signalling WikiPathway driven by the *DOK5* gene. There were many miRNA enrichments all driven by the *FOXN3* gene.

When we investigated functional gene enrichments across time for PHQ8 and PTSD at 3-, 6-, and 12-months post-stroke, we observed that these measures consistently showed enrichment of the *CTNNA1–CTNNB1* complex and cell*–*cell junction complex (*ARHGAP10–CTNNA1*) CORUM terms.

#### Enrichment results from PPI networks

Next, we created PPI networks for each phenotype to determine the extent of enrichment overlap between them. The PPI networks were enriched for GO terms including nervous system development (SIS-ADL at 3 months), post-synaptic density (PHQ8 at 12 months), neuron projection (tMOCA), axon guidance (delta grip strength 1–3 months). Supplementary File 2 contains PPI for multiple phenotypes and their GO term enrichments, demonstrating the consistency of biological mechanisms consistent with neural plasticity. The most frequent were related to synapse or cell junction biology across four uncorrelated phenotypes: delta grip strength at 3 months, SIS-ADL at 3 months, PHQ8 at 12 months and tMoCA at 12 months (Supplementary Fig. 2A–D).

The most enriched PPI network was for SIS-ADL scores at 3 months, which had 99 proteins and 42 edges (online interactive figure) and was significantly enriched for PPI at *P* = 3.42e-5, demonstrating that our set of query proteins had more interactions than would be expected for a random set of proteins drawn from the genome of the same size and degree distribution (expected edges = 21).

The overall interaction network for SIS-ADL scores showed significant enrichment (FDR < 0.05) in several GO terms. The strongest enrichment effects [log10(observed/expected)] were for the GO biological process terms activation of transmembrane receptor protein tyrosine kinase activity (FDR = 0.015), regulation of cell junction assembly (FDR = 0.0199) and nervous system development (FDR = 0.0174).

## Discussion

Substantial variability exists in stroke outcomes that is not completely explained by clinical and neuroimaging measures.^6^ For example, the NIHSS total score 5–10 days post-stroke only explains 15% of the variance of the NIHSS total score at 3 months in the STRONG cohort.^17^ Genetic studies may be useful to gain insights into this substantial unexplained variance in stroke recovery.^12,14^ Notably, most genetic studies of stroke recovery have focused on candidate gene approaches, which require significant prior knowledge and accuracy of gene selection. Hence, these candidate gene approaches have limitations for discovery. Other studies made progress in genetic discovery for stroke recovery by using genome-wide association approaches,^10-12^ but lacked replication and suffered from using global outcome measures (e.g. mRS score) that combine multiple behavioural domains into a single value, which obfuscates recovery measurement. Recently, our group adapted the NIHSS and utilized its sub-items to measure the cognitive and motor burden of stroke and their rate of change over time using GWAS.^13,14^ However, these deficit-specific adaptations, the NIH Cog-4 (refs ^14, 41^) and NIH Motor-6,^14^ remain crude, with significant ceiling effects compared to more granular measures (e.g. SIS-ADL or grip strength).

The current study aimed to advance stroke recovery genomics by performing genome-wide associations of several granular, specific stroke recovery behavioural domains over multiple time points. We generated nine sets of stroke recovery GWAS summary statistics, which can further be used by the scientific community. We observed 319 cross-phenotypic SNPs, even after accounting for the correlation structure among phenotypes, and many of these genes are relevant to neural development, function and plasticity. These results suggest that there may be genes that influence stroke recovery in a broad way.

### Cross-phenotypic genome-wide associations

Our analytical approach allowed for the discovery of cross-phenotype genetic associations, which show pleiotropy of specific SNPs pointing to shared biological mechanisms and pathways, especially when the phenotype measures are uncorrelated (Figs 1 and 2). We lacked any cross-phenotypic SNP with a *P-*value < 5e-8 across at least two phenotypes. However, we found 319 SNPs at the suggestive level (*P* < 5e-5) among two or more phenotypes. These SNPs were in biologically relevant genes, including *FOXN3, ERICH1, LIF-AS1, RPS6KA2* and *TUBGCP2*.

*FOXN3* acts as a transcriptional repressor involved in minimizing DNA damage^38^ and has a role in glucose metabolism, and was shared across stress (PTSD) and cognition (tMoCA) at 12 months.^39,40^  *FOXN3* appears to be involved in insulin sensitivity in individuals with and without type 2 diabetes mellitus,^42^ likely through regulation of fasting glucose levels.^43^ Fasting blood glucose levels are positively correlated with stroke risk.^44^ Fasting blood glucose control appears to influence post-stroke outcomes by 6 months,^45^ but not by 3 months.^46^

Regarding *ERICH1*, SNP (rs11778883-C) is associated with *PQBP1*, *NEGR1*, *CDNF* and *PGK2* protein levels (*P* < 0.001; see Supplementary Table 3), and was shared across depression (PHQ8 at 6 months) and cognition (tMoCA at 12 months).^33^ These are important proteins in the context of neurological disease. For example, *CDNF*, in conjunction with mesencephalic astrocyte-derived neurotrophic factor, has neuroprotective effects against cerebral ischaemia in animal studies.^47,48^ Other studies suggest that *CDNF* promotes functional recovery by mediating acute stroke inflammation,^49^ without minimizing post-stroke neuronal loss.^50^ As a potential therapeutic, CDNF has been demonstrated to be safe and well-tolerated in patients with Parkinson’s disease.^51^

*LIFR-AS1* is an antisense RNA gene to the sense gene Leukemia Inhibitory Factor Receptor (*LIFR)*, also known as *CD118*, and was shared across motor (delta grip) and stress (PTSD at 12 months). *LIFR* is involved in multiple pathways including MAPK signalling and JAK/STAT3.^52^ In a middle cerebral artery occlusion model,^53^  *LIFR* protein expression in brain tissue was under-expressed in aged rodents compared to young ones. This led to the reduction of the neuroprotective effect of leukaemia inhibitory factor to promote post-stroke motor improvement. In a multi-omics study,^54^  *LIFR* genetic susceptibility upregulation was associated with the reduced risk of large artery stroke. They did not investigate if *LIFR* had an association with stroke recovery measures. It is unknown how *LIFR-AS1* genetic variants affect function, structure or the ability to regulate *LIFR* expression.

*RPS6KA2* is a serine/threonine-protein kinase, which is overexpressed in adult brain tissue, especially the prefrontal cortex, and was shared across depression (PHQ8) and functional status (SIS-ADL) at 3 months.^55^ It appears to affect cell differentiation via the MAPK/STAT3 signalling pathway (KEGG pathway hsa04010).^56^ Several cross-phenotypic SNPs between PHQ8 and PTSD at 12 months post-stroke also had pQTL associations with STAT3 protein levels. Additionally, *RPS6K2’s* protein has been found to be druggable based on the druggable gene dataset from Finan *et al.*^57^; druggable genes are genes that encode proteins that have the capability of being modulated by drug-like molecules. In fact, a selective ribosomal S6 kinase inhibitor (SL 0101-1) is used in animal models for cancer research to understand the impact of MAPK/STAT3 signalling on cancer cell growth.^58^ The current results suggest utility in examining the translation potential of *RPS6K2*’s for stroke recovery in animal models. Furthermore, current *RPS6K2* formulations could be tested for safety and tolerance through dose-escalation studies.

*LOC105371908* was shared across motor (delta grip) and cognition (tMoCA at 12 months) via rs149456987-T (Supplementary Table 1). It is a long non-coding RNA. This suggests that *LOC105371908* has a regulator effect. The gene is located ∼4.5 kb downstream of a gene enhancer region (GH17J077836). The gene enhancer region has transcription factor binding sites for *CEBPB*, *CEBPG*, *FOXK2*, *GLIS1*, *HLF*, *NFIC*, *NR2F1*, *ZBTB40*, *ZNF184* and *ZNF600*. Some of these genes play an important role in RNA polymerase II transcription regulator complex.^59,60^ Thus, it may have a regulator effect on the RNA polymerase II transcription regulator complex, but there is no direct evidence to confirm this. The effect of rs149456987-T is in the same direction for both phenotypes, suggesting a consistency of effect. As such, rs149456987-T is a candidate biomarker for future validation studies.

The rs149456987-T SNP is within the *TUBGCP2* (Tubulin Gamma Complex Component 2) gene, is involved in brain development as well as neuronal migration, and plays a major role in neuronal survival by inhibiting apoptosis.^61,62^ It is associated with microcephaly and neurodevelopmental disease^61,63^ by altering the structure of the gamma-tubulin ring complex (y-TuRC). The gamma-tubulin encoded by *TUBG2* is a key component of the y-TuRC^26^ and is mainly expressed in the brain.^64^ While *TUBG2* is druggable,^57^ its mechanism of action within brain tissue is unknown, and the potential utility of a drug targeting *TUBG2* and/or y-TuRC may depend on its ability to cross the blood-brain barrier.

### Protein–protein interaction networks and enrichment analyses

We generated PPI networks separately for each of the nine stroke recovery phenotypes to preserve domain-specific biology and ensure enough signal density to identify coherent modules and hub proteins, then compared networks/enrichments across phenotypes to detect shared themes. Only the SIS-ADL genetic associations resulted in a stable PPI network. *EFNA5* (Ephrin-A5), a hub node in this network, is a cell surface-bound ligand of several Ephrin receptor tyrosine kinases that are crucial for cell migration, repulsion and adhesion during neuronal, vascular and epithelial development,^30^ and is also expressed by developing spinal motor neurons.^31^ The *EFNA5* cluster in the SIS-ADL network also includes *SLIT3* (Slit homolog 3 protein), which acts as molecular guidance cue in cellular migration^30^ and is co-expressed with *EFNA5*.^32^  *SLIT3* is part of the SLIT–ROBO complex that regulates axon guidance.^65^ This is concordant with our prior work, in an independent cohort, which found SNP associations with motor and global post-stroke recovery within the *ROBO1* and *ROBO2* genes.^14^

In contrast, the cross-phenotype SNPs mapped to relatively few protein-coding genes and produced sparse, weakly interconnected PPI graphs with limited GO enrichment, suggesting that cross-domain overlap may reflect shared regulatory/non-coding mechanisms upstream of direct protein–protein interactions; accordingly, we used cross-phenotype functional enrichment alongside within-phenotype PPI construction to capture both shared and domain-specific recovery biology. Gene enrichment of all cross-phenotypic SNPs had associated terms including ‘*CREB1* phosphorylation through NMDA receptor-mediated activation of RAS signalling’ and ‘Signalling by neurotrophin tyrosine kinase receptors (*NTRKs*)’ REAC terms. *CREB1* (cAMP Response Element Binding Protein 1) is known to control post-stroke cortical circuit plasticity and functional recovery in mouse models, especially when activated in the initial stages of cell death.^66^ Furthermore, *CREB1* activity modulates *BDNF* and its resulting *NTKR* signalling in cortical neurons, which is highly conserved between mice and humans.^67^  *BDNF* and *NTKR1* & *2* are druggable as well.^57^

Both *EFNA5* and *SLIT3*, along with two other proteins in this cluster (*EPHA1* and *GRIK1*) are annotated with GO terms for nervous system development. *EFNA5*, *PTPRD*, *PTPRS* and *THBS2* are annotated with GO terms for regulation of synapse assembly. Additionally, there was consistency of GO terms related to nervous system development and post-synaptic density across delta grip and SIS-ADL at 3 months and PHQ8 at months, as well as to neuron projection and axon guidance across the delta grip at 3 months and tMoCA at 12 months (Supplementary File 2). We believe that this consistency among the PPI networks may indicate either (1) an influence by these genes on neuronal and vascular guidance signalling during brain development as determinants of resilience and inherent post-stroke recovery potential (e.g. latent neural pathways or vascular collaterals) or (2) alternatively, this association might reflect the role these genes have in activity-dependent plasticity as part of a stroke recovery mechanism.

### Shared architecture between anxiety and depression

We observed moderate correlation between PTSD and PHQ8 across 6- and 12-month post-stroke timepoints (Fig. 3). And we had the same enrichment terms between cross-phenotypic SNPs of all PTSD phenotypes and cross-phenotypic SNPs of all PHQ8 phenotypes (Supplementary Table 5). This remained true with enrichment results of PTSD and PHQ8 phenotypes at the same timepoint (e.g. 6 months post-stroke). Based on this, we believe that the depression and stress domains demonstrate a shared psychopathology. As a result, the suggestive genetic variants shared between depression and stress are likely reflecting the same genetic architecture.

### Comparison to other methods

The cross-phenotypic approach implemented in this exploratory study relies on phenotypes being uncorrelated (i.e. independent). Furthermore, this approach was tailored to the STRONG study’s clinical and genotype data. However, there are other methods that model multiple traits (aka phenotypes) directly within a GWAS framework, like the statgenQTLxT R package.^68,69^ The strength of this approach is the direct modelling of covariance across phenotypes. Another method is an omnibus test that combines *P*-values across GWAS summary data sets. The ACAT R package implements an omnibus test of *P*-values for all shared SNPs among tested data sets, which is robust to dependence among phenotypes.^70,71^  Supplementary File 3 contains a description of the implementation, rationale and results of both methods. In brief, the statgenQTLxT software does not allow missing data, which decreased the sample size to 200 individuals with all nine phenotypes observed, which is more than a 50% reduction in sample size. This led to notable modelling artefacts (Supplementary Fig. 3A and B). The omnibus approach suffered from smaller *P*-values overshadowing larger ones (Supplementary Fig. 3C). For this study, the statgenQTLxT and ACAT methods had notable issues suggesting that our cross-phenotypic approach is preferred.

### Limitations

This study has limitations. The first is notable genomic inflation of *P-*values in the delta grip genome-wide associations above the 75th percentile based on the QQ-plot (Supplementary Fig. 1B), despite an acceptable lambda value of 1.02 (Table 3). This leads to an inflation of small *P-*values below the suggestive (*P* 5e-5) and significant (*P* 5e-8) thresholds. However, our cross-phenotype approach mediated this issue. Another limitation is the lack of statistical power due to low sample sizes. Therefore, we have an elevated rate of false negative associations. However, the cross-phenotypic SNP count was significantly higher than expected due to chance, suggesting that incorporating multiple GWASs related to distinct phenotypes may increase statistical power by leveraging joint probabilities. There is a caveat. Our cross-phenotypic approach assumes independences between phenotypes that are part of the same disease, disorder, or exposure. That can be a strong assumption, while other methods seek to account for the inter-phenotype correlation structure directly or with SNP *P-*values across studies. Methods using SNP *P-*values across studies rely on well-powered GWAS with sample sizes over ten thousand, which limits their application to smaller GWASs conducted in burgeoning fields like stroke recovery. Even though the alternative approaches we explored had issues, they were not tested extensively with simulations where the ground truth is known against the cross-phenotypic approach. Therefore, one cannot conclude any superiority of the cross-phenotypic approach to the statgenQTLxT and ACAT methods.

## Conclusions

This exploratory study adds to the list of post-stroke recovery genes that can be studied as biomarkers or as therapeutic targets. This study utilized the deep phenotyping offered by the STRONG study^17^ to conduct nine novel stroke recovery phenotype GWAS; now available to the scientific community. We replicated the *SLIT-ROBO* axon guidance mechanism association from a prior post-stroke recovery GWAS^14^ within a PPI network from the SIS-ADL GWAS. We also observed several cross-phenotypic gene associations, including the *ERICH1*, *FOXN3*, *LIFR-AS1*, *RPS6KA2 and TUBGCP2* genes. Some of these genes show potential as biomarkers because they influence developmental effects, suggesting vulnerability or resilience to stroke. Others code for proteins that may be druggable and may have therapeutic implications for stroke recovery. Additionally, we observed functional cross-phenotypic gene enrichment of *CREB1* phosphorylation, a known driver of post-stroke plasticity and recovery. Our cross-phenotypic approach may be useful for future genomic studies in which enrolling large numbers of participants is not feasible.

## Supplementary Material

fcag322_Supplementary_Data

## Data Availability

The GWAS summary statistics for the nine phenotypes can be found at the Cerebrovascular Disease Knowledge Portal (https://cd.hugeamp.org/) where they can be downloaded. Code generated for this study can be found at the GitHub repository https://github.com/CMAldridge/Cross-Phenotype-GWAS upon publication. STRONG clinical and genotype individual-level data will be released upon reasonable request to the corresponding author, S.C.C. In the future, the clinical and genotyped data will be available at the NIH’s dbGaP platform.
