# Supplementary material for: Genomic insights into stroke recovery: cross-phenotype associations
Source: Brain Commun. 2026 Aug 26;8(5):fcag322. doi: 10.1093/braincomms/fcag322 (PMC13540738; doi:10.1093/braincomms/fcag322)
Supplement: Supplementary file 1 [file fcag322_supplementary_data.zip › Supplementary File 1.docx]

Supplementary Figure 1A-Manhattan plot of Delta Grip Ratio at 3 months post-stroke. The red line is genome-wide significant. Gene names are tied to the top significant SNP within a given genomic locus. P values derived from t-statistics calculated by linear regression models.

**Lambda 1.02**

Supplementary Figure 1B - Delta Grip at 3 months post-stroke QQ plot. Lambda is a measure of genomic inflation; a normal value is 1.05 or less. P values derived from t-statistics calculated by linear regression models.

Supplementary Figure 1C - Manhattan plot of the mRS at 3 months post-stroke. The red line is genome-wide significant. Gene names are tied to the top significant SNP within a given genomic locus. P values derived from F-statistics calculated by logistic regression models.

**Lambda 1.01**

Supplementary Figure 1D - QQ plot of the mRS 0-2 vs 3-6 at 3 months post stroke. A normal lambda value is 1.05 or less. P values derived from F-statistics calculated by logistic regression models.

Supplementary Figure 1E - Manhattan plot of the PHQ8 at 3 months post-stroke. The red line is genome-wide significant. Gene names are tied to the top significant SNP within a given genomic locus. The PHQ8 is a measure of depression. P values derived from t-statistics calculated by linear regression models.

**Lambda 1.01**

Supplementary Figure 1F - QQ plot of PHQ8 at 3 months post-stroke. A normal lambda value is 1.05 or less. P values derived from t-statistics calculated by linear regression models.

Supplementary Figure 1G - Manhattan plot of the SIS-ADL at 3 months post-stroke. The red line is genome-wide significant. Gene names are tied to the top significant SNP within a given genomic locus. SIS-ADL is the Stroke Impact Scale Activities of Daily Living total score. P values derived from t-statistics calculated by linear regression models.

**Lambda 1.02**

Supplementary Figure 1H - QQ plot of the SIS-ADL score at 3 months post-stroke. A normal lambda value is 1.05 or less. SIS-ADL is the Stroke Impact Scale Activities of Daily Living total score. P values derived from t-statistics calculated by linear regression models.

Supplementary Figure 1I - Manhattan plot of the PHQ8 at 6 months post-stroke. The red line is genome-wide significant. Gene names are tied to the top significant SNP within a given genomic locus. PHQ8 is a measure of depression. P values derived from t-statistics calculated by linear regression models.

**Lambda 1.00**

Supplementary Figure 1J - QQ plot of the PHQ8 at 6 months post-stroke. A normal lambda value is 1.05 or less. PHQ8 is a measure of depression. P values derived from t-statistics calculated by linear regression models.

Supplementary Figure 1K - Manhattan plot of the PTSD at 6 months post-stroke. The red line is genome-wide significant. Gene names are tied to the top significant SNP within a given genomic locus. PTSD is the Primary Care PTSD measure of anxiety. P values derived from t-statistics calculated by linear regression models.

**Lambda 1.05**

Supplementary Figure 1L - QQ plot of the PTSD score at 6 months post-stroke. A normal lambda value is 1.05 or less. PTSD is the Primary Care PTSD score, a measure of anxiety. P values derived from t-statistics calculated by linear regression models.

Supplementary Figure 1M - Manhattan plot of the PHQ8 at 12 months post-stroke. The red line is genome-wide significant. Gene names are tied to the top significant SNP within a given genomic locus. PHQ8 is a measure of depression. P values derived from t-statistics calculated by linear regression models.

**Lambda 1.02**

Supplementary Figure 1N - QQ plot of the PHQ8 at 12 months post-stroke. A normal lambda value is 1.05 or less. P values derived from t-statistics calculated by linear regression models.

Supplementary Figure 1O - Manhattan plot of the PTSD measure at 12 months post-stroke. The red line is genome-wide significant. Gene names are tied to the top significant SNP within a given genomic locus. PTSD the Primary Care PTSD measure of anxiety. P values derived from t-statistics calculated by linear regression models.

**Lambda 1.03**

Supplementary Figure 1P - QQ plot of the PTSD measure at 12 months post-stroke. A normal lambda value is 1.05 or less. P values derived from t-statistics calculated by linear regression models.

Supplementary Figure 1Q - Manhattan plot of tMoCA score at 12 months post-stroke. The red line is genome-wide significant. Gene names are tied to the top significant SNP within a given genomic locus. tMoCA is the telephone version of the Montreal Cognitive Assessment total score; as measure of cognition. P values derived from t-statistics calculated by linear regression models.

**Lambda 1.03**

Supplementary Figure 1R - QQ plot of the tMoCA score at 12 months post-stroke. A normal lambda value is 1.05 or less. tMoCA is the telephone version of the Montreal Cognitive Assessment total score; as measure of cognition. P values derived from t-statistics calculated by linear regression models.
