# Supplementary material for: Genomic insights into stroke recovery: cross-phenotype associations
Source: Brain Commun. 2026 Aug 26;8(5):fcag322. doi: 10.1093/braincomms/fcag322 (PMC13540738; doi:10.1093/braincomms/fcag322)
Supplement: Supplementary file 1 [file fcag322_supplementary_data.zip › Supplementary_File_2.docx]

## Protein-Protein Interaction Networks and Enrichment Analyses

PPI networks for several phenotypes were significantly enriched in protein-protein interactions, and for GO terms that included Nervous System Development (SIS ADL at 3 mos), Postsynaptic Density (PHQ8), Post-Synapse (tMOCA), and Axon guidance (change in Grip Strength from 1-3 mos.).

### SIS ADL Scores

The PPI network for SIS ADL scores at 3 months (Figure online at [SIS ADL 90d Network Plot](https://www.ndexbio.org/#/network/9911bdb1-2b50-11f0-a469-005056ae3c32?accesskey=c30a335e9f2fd41e8a3a5998f8739a801b1f8845ab9bad63f685943d2cedc197)), with 99 proteins and 42 edges, was significantly enriched for protein-protein interactions at p = 3.42E-5, demonstrating that our set of query proteins had more interactions than would be expected for a random set of proteins drawn from the genome of the same size and degree distribution (expected edges = 21). The overall interaction network for SIS-ADL scores showed significant enrichment (FDR <0.05) in several GO terms as depicted in **Supplementary** **Figure 2A**. The strongest enrichment effects were for the GO Biological Process terms Activation of transmembrane receptor protein tyrosine kinase activity, Positive regulation of cell junction assembly, and Nervous system development. The strength measure (Log10 (observed/expected)) describes how large the enrichment effect is as the ratio between i) the number of proteins in the network that are annotated with a term and ii) the number of proteins in a random network of the same size that would be expected to be annotated with this term.

**Supplementary Figure 2A**

Bubble plot of Gene Ontology (GO) Biological Process term enrichment for the SIS ADL Protein-Protein Interaction Network. GO terms are on the y-axis, and enrichment strength is on the x-axis. Bubble size indicates gene count, and bubble color indicates the false discovery rate (FDR). For this analysis, n = 99 protein-coding genes were evaluated. Statistical significance for GO term enrichment was computed using a hypergeometric test and corrected for multiple testing using the Benjamini and Hochberg method, with significance reported as FDR-adjusted p values. The plotted values correspond to the indicated FDR ranges.

### PHQ8 Scores at 12 Months

The PPI network for PHQ8 scores (Figure online at [PHQ8 Network Plot](https://www.ndexbio.org/viewer/networks/af9fd1c3-2b50-11f0-a469-005056ae3c32)), with 87 proteins and 39 edges, was significantly enriched for protein-protein interactions (p = 0.007, expected edges = 25). The overall interaction network for PHQ8 scores showed significant enrichment in GO Cellular Component terms as depicted in **Supplementary** **Figure 2B**, the strongest of which was Postsynaptic Density.

**Supplementary Figure 2B**

Bubble plot of Gene Ontology (GO) Cellular Component term enrichment for the PHQ8 Protein-Protein Interaction Network. GO terms are on the y-axis, and enrichment strength is on the x-axis. Bubble size indicates gene count, and bubble color indicates the false discovery rate (FDR). For this analysis, n = 87 protein-coding genes were evaluated. Statistical significance for GO term enrichment was computed using a hypergeometric test and corrected for multiple testing using the Benjamini and Hochberg method, with significance reported as FDR-adjusted p values. The plotted values correspond to the indicated FDR ranges.

### tMOCA Scores

The PPI network for tMOCA scores (Figure online at [tMOCA Network Plot](https://www.ndexbio.org/viewer/networks/c6958145-2b50-11f0-a469-005056ae3c32)), with 136 proteins and 98 edges, was not significantly enriched for known protein-protein interactions (p = 0.18, expected edges 89). Nonetheless the 136 protein-coding genes associated with tMOCA scores on our GWAS did show significant enrichment in several GO Cellular Component terms as depicted in **Supplementary** **Figure 2C**, the strongest of which were Extrinsic component of membrane, Post-synapse, Anchoring junction and Synapse.

**Supplementary Figure 2C**

Bubble plot of Gene Ontology (GO) Cellular Component term enrichment for the tMoCA Protein-Protein Interaction Network. GO terms are on the y-axis, and enrichment strength is on the x-axis. Bubble size indicates gene count, and bubble color indicates the false discovery rate (FDR). For this analysis, n = 136 protein-coding genes were evaluated. Statistical significance for GO term enrichment was computed using a hypergeometric test and corrected for multiple testing using the Benjamini and Hochberg method, with significance reported as FDR-adjusted p values. The plotted values correspond to the indicated FDR ranges.

### Change in Grip Strength

Because the large number genetic loci associated with grip strength changes in our GWAS raises the risk of false positives, we constructed the PPI network for this phenotype with a more stringent confidence threshold (0.7 = high vs. 0.4 = moderate in the other networks reported here). The resulting network for this phenotype (Figure online at [Grip Strength Change Network](https://www.ndexbio.org/viewer/networks/d7d73ac8-2b50-11f0-a469-005056ae3c32)), with 601 proteins and 245, edges was significantly enriched for protein-protein interactions (expected edges 158, p = 1.01E^-10^). The overall interaction network for the change in grip strength showed significant enrichment in several GO Biological Process terms as depicted in **Supplementary** **Figure 2D**, the strongest of which were Heterophilic cell adhesion, Axon guidance, and Telencephalon development. GO Cellular Component terms were also significantly enriched, the strongest of which were Neuronal cell body membrane, Basement membrane and Neuron to neuron synapse as shown in **Supplementary** **Figure 2E**.

**Supplementary Figure 2D**

Bubble plot of Gene Ontology (GO) Biological Process term enrichment for the change in grip strength Protein-Protein Interaction Network. GO terms are on the y-axis, and enrichment strength is on the x-axis. Bubble size indicates gene count, and bubble color indicates the false discovery rate (FDR). For this analysis, n = 601 protein-coding genes were evaluated. Statistical significance for GO term enrichment was computed using a hypergeometric test and corrected for multiple testing using the Benjamini and Hochberg method, with significance reported as FDR-adjusted p values. The plotted values correspond to the indicated FDR ranges.

**Supplementary Figure 2E**

Bubble plot of Gene Ontology (GO) Cellular Component term enrichment for the Protein-Protein Interaction Network. GO terms are on the y-axis, and enrichment strength is on the x-axis. Bubble size indicates gene count, and bubble color indicates the false discovery rate (FDR). For this analysis, n = 601 protein-coding genes were evaluated. Statistical significance for GO term enrichment was computed using a hypergeometric test and corrected for multiple testing using the Benjamini and Hochberg method, with significance reported as FDR-adjusted p values. The plotted values correspond to the indicated FDR ranges.
