# Supplementary material for: Genomic insights into stroke recovery: cross-phenotype associations
Source: Brain Commun. 2026 Aug 26;8(5):fcag322. doi: 10.1093/braincomms/fcag322 (PMC13540738; doi:10.1093/braincomms/fcag322)
Supplement: Supplementary file 1 [file fcag322_supplementary_data.zip › Supplementary_File_3.docx]

We decided to utilize two methods that we now discuss in the introduction section as comparators to our design. First, we implemented the statgenQTLxT R package method.^1,2^ The strength of this approach is directly modelling the individual level data across multiple phenotypes from the same cohort/study. Their approach also estimates the covariance structure for adjustment of correlated phenotypes. However, this software does not allow any missing data in the phenotype data set. We observed a complete analysis data set of 201 participates with complete data across the nine phenotypes, which is a loss of over half of the sample size.

That said, we found a 10,301 genome significant hits, but with large genome inflation (1.14). After genomic control was applied, no genome-wide significant hits remained. This is likely due to the notable drop in sample size, leading to significant right p-value tail inflation due to SNPs with low minor allele frequencies; see Supplementary Fig. 3A and 3B. We also observed no overlap of our cross-phenotype primary findings and any suggestive (p<5e-5) SNPs from the multi-trait GWAS with genomic control.

Supplementary Figure 3A - Manhattan Plot from statgenQTLxT. This figure shows extremely small p values on the -log10 scale from modeling issues. These single nucleotide polymorphisms (SNPs) had less than a 5% minor allele frequency in the genotyped data set. P values were derived from likelihood ratio (F) tests based comparing a null model with the multivariate models iteratively including SNPs.

Supplementary Figure 3B - Quantile-Quantile plot. The observed -log10(p values) are greatly inflated due to modeling artifact. This mirrors the Manhattan plot (Supplementary Fig. 3A) above. P values were derived from likelihood ratio (F) tests based comparing a null model with the multivariate models iteratively including SNPs.

The second method, we implemented the ACAT R package omnibus test approach.^3,4^ This approach combines SNP p-values across multiple GWAS summary data sets. The ACAT R package implements the **Aggregated Cauchy Association Test**, a fast, analytic p‑value combination method that is widely used in rare-variant and omnibus genetic association testing.

However, this approach performed poorly when we tested it on the GWAS summary statistics from Delta Grip and tMoCA. Supplementary Fig. 3C below shows how the Delta Grip p-values (panel B) completely overwhelm the tMoCA ones (panel C) in the combined p values (panel A). This approach does not address any right tail inflation of very small p-values, like our heuristic.

Supplementary Figure 3C - Stacked Manhattan plots of (A) Combined P-values, (B) Delta Grip, and (C) tMoCA. Combined p-values are the resulting p-values from the ACAT R package's omnibus test. Delta grip (v2_grip_change; Middle Manhattan plot) is the change in grip ratio from 2 to 10 days minus ratios at 3 months post-stroke. V4_tMocA is the telephone Montreal Cognitive Assessment at 12 months post-stroke (bottom Manhattan plot). The red dots with rsIDs are the top single nucleotide polymorphisms (SNPs) of genetic loci from the combined p-values derived from the omnibus test. Panels B and C derived their p values from their respective linear regression genome-wide association t-statistics.

**References**

1.	Zhou, X. & Stephens, M. Efficient multivariate linear mixed model algorithms for genome-wide association studies. *Nat. Methods* **11**, 407–409 (2014).

2.	Theoretical background.

3.	Liu, Y. *et al.* ACAT: A Fast and Powerful p Value Combination Method for Rare-Variant Analysis in Sequencing Studies. *Am. J. Hum. Genet.* **104**, 410–421 (2019).

4.	Liu, W., Guo, Y. & Liu, Z. An Omnibus Test for Detecting Multiple Phenotype Associations Based on GWAS Summary Level Data. *Front. Genet.* **12**, (2021).
